# Preoperative CT displacement predicts inferior alveolar nerve injury in mandibular fractures: a tool for clinical risk stratification

**DOI:** 10.1007/s00784-026-07002-7

**Published:** 2026-07-17

**Authors:** Lucas M Ritschl, Bastian Schwaiberger, Katharina Pippich, Jannik Ketschau, Victoria Kehl, Wolff Klaus-Dietrich, M Probst, Andreas Fichter, Jonathan Mohr

**Affiliations:** 1 Department of Oral and Maxillofacial Surgery, School of Medicine and Health, TUM University Hospital Klinikum Rechts der Isar, Technical University of Munich, Ismaninger Strasse 22, D-81675 Munich, Germany; 2 Department of Neuroradiology, School of Medicine and Health, TUM University Hospital Klinikum Rechts der Isar, Technical University of Munich, Munich, Germany; 3 Institute of AI and Informatics in Medicine, School of Medicine and Health, TUM University Hospital Klinikum Rechts der Isar, Technical University of Munich, Munich, Germany; 4 Department of Oral and Maxillofacial Surgery, University Hospital Leipzig, Leipzig, Germany

**Keywords:** Mandibular fractures, Inferior alveolar nerve (IAN) injury, Computed tomography, Cortical displacement, Mandibular canal involvement, Sensory deficits, Predictive classification

## Abstract

**Objectives:**

The aim of this retrospective study was to determine the incidence of inferior alveolar nerve (IAN) injury in patients with mandibular fractures by correlating fracture displacement and mandibular canal involvement on preoperative computed tomography (CT) imaging with clinically documented nerve deficits – and to develop the first CT-based displacement classification for predicting the likelihood of IAN injury.

**Materials and methods:**

We retrospectively reviewed mandibular fractures and available preoperative CT scans. Fracture location, cortical and canal displacement, and nerve function were recorded. Receiver operating characteristic (ROC) curve and logistic regression analyses evaluated associations between displacement thresholds and IAN injury risk.

**Results:**

A total of 208 patients (237 fractures) were included; preoperative IAN deficits occurred in 74/237 fractures (31.2%) and persisted at ≥ 6 months in 3/237 fractures (1.3%). ROC analysis yielded area under the curve (AUC) values of 0.71, 0.73, 0.75, and 0.75 for cortical displacement cut-offs of > 3.60 mm (transverse), > 1.68 mm (sagittal), > 1.29 mm (vertical), and > 9.61 mm (summed displacement), respectively. Multivariate logistic regression demonstrated that exceeding these thresholds was associated with significantly increased risk of IAN hypoesthesia – OR 6.4, 4.8, 6.8, and 7.9, respectively (all *p* < 0.001). Mandibular canal displacement showed limited predictive value for IAN hypoesthesia, with ROC AUCs of 0.63 (axial), 0.63 (coronal), 0.64 (sagittal), and 0.64 (summed displacement).

**Conclusion and Clinical Relevance:**

This study establishes specific, plane-based CT displacement thresholds that may help to identify mandibular fractures at high risk of IAN injury. Our proposed CT-based classification enables objective preoperative risk stratification, enhances patient counseling, guides surgical decision-making, and supports the standardized medico-legal assessment of potential permanent sensory deficits.

## Brief summary

This study retrospectively analyzed 208 patients with 237 mandibular fractures at a tertiary care center. It aimed to correlate preoperative computed tomography (CT) measured cortical displacement and mandibular canal involvement with clinically documented inferior alveolar nerve (IAN) deficits and to develop a CT-based classification for predicting IAN hypoesthesia based on displacement severity. Preoperative IAN deficits were observed in 31.2% of fractures, with persistent deficits at ≥ 6 months in 1.3%. Receiver operating characteristic (ROC) analysis determined displacement thresholds with the highest predictive accuracy – transverse > 3.60 mm (AUC 0.71), sagittal > 1.68 mm (AUC 0.73), vertical > 1.29 mm (AUC 0.75), and summed > 9.61 mm (AUC 0.75). Multivariate logistic regression demonstrated that exceeding these thresholds was associated with a 6.4- to 7.9-fold increased risk of IAN hypoesthesia (all *p* < 0.001). The resulting classification provides a practical tool for surgical planning, patient counseling, and medico-legal expert assessments of permanent nerve injury risk.

## Introduction

The epidemiology of facial fractures has changed substantially in recent decades, reflecting socio-economic development, demographic aging, and region-specific shifts in trauma mechanisms. Accordingly, studies report heterogeneous trends, including increasing absolute case numbers but decreasing age-standardized incidence rates. Nevertheless, 10–15% of all trauma patients sustain injuries to the oral and maxillofacial region [1–4]. 

The mandible, due to its morphology and exposed position within the viscerocranium, is particularly prone to fracture [3, 5], making mandibular fractures among the most common facial skeletal injuries after nose and orbito-zygomatic injuries [6, 7]. The etiology of mandibular fractures varies by mechanism: Traffic-related injuries now account for only 10–12% of cases [8], while leisure and sports accidents represent approximately 43% of viscerocranial fractures [9]. However, assaults remain the leading cause of mandibular fractures, with reported rates ranging from 28 to 84.5% [10–13]. 

The proximity of the mandible to the peripheral trigeminal branches – the inferior alveolar and lingual nerves – renders these nerves vulnerable in mandibular fractures; inferior alveolar nerve (IAN) lesions, in particular, are far more common than lingual nerve injuries [14]. The IAN, coursing through the mandibular canal, is predominantly stretched or compressed in fractures between the mandibular and mental foramina, whereas complete nerve transections remain rare. Injury mechanisms include indirect trauma to the neurovascular bundle, compression from soft-tissue edema, and direct involvement within the fracture line via displacement, traction, or compression [13]. Inferior alveolar nerve injury is a well-characterized complication of mandibular fractures, with numerous studies documenting its prevalence both at initial presentation and following surgical management.

Summarizing, IAN hypoesthesia occurs in up to 40% of mandibular fracture cases at presentation, a figure that may increase following surgical intervention, underscoring the importance of accurate preoperative risk stratification based on displacement measurements [15–19]. 

Multiple authors identified a 5 mm fracture gap on two-dimensional panoramic radiographs as the threshold above which IAN recovery rates declined significantly. However, these studies did not differentiate displacement by anatomical plane or utilize three-dimensional imaging [20–23]. Moreover, Tay et al. (2015) demonstrated that each additional millimeter of fracture gap further increases the odds of post-treatment IAN hypoesthesia by approximately 27% [24]. It is also known that fractures displaced by more than 9 mm exhibit persistently poor IAN recovery [22, 25]. 

To date, no displacement-based CT classification exists for predicting IAN hypoesthesia in mandibular fractures. Therefore, the aim of this study was to evaluate the association between preoperative CT-measured fracture displacement and clinically documented inferior alveolar nerve (IAN) deficits. We hypothesized that increasing three-dimensional cortical displacement is associated with a significantly higher risk of IAN hypoesthesia. In addition, we aimed to develop a clinically applicable CT-based classification system for objective preoperative risk stratification.

## Materials and methods

### Ethical statement and study collective

All clinical investigations were conducted in accordance with the Declaration of Helsinki and were approved by the institutional ethics committee of the Technische Universität München, Klinikum rechts der Isar (File No. 429/18 S-KK).

This retrospective analysis initially encompassed all patients admitted for treatment of mandibular fractures at the Department of Oral and Maxillofacial Surgery, TUM University Hospital Klinikum Rechts der Isar, School of Medicine and Health, Technical University of Munich, Germany, from January 1, 2013, to December 31, 2017. Patients were identified via ICD-10-GM codes S02.60–S02.69. Only fractures localized between the mandibular foramen and mental foramen were included. Eligible cases were those managed as inpatients and with available preoperative three-dimensional CT imaging stored in the hospital information system.

Exclusion criteria included metabolic bone diseases, osseous metastases, head-and-neck malignancies, antiresorptive therapy, and neurological disorders (except epilepsy). Additional study-specific exclusions comprised pathological fractures, fractures outside the defined anatomical region, and atrophic mandibles with a residual height < 15 mm.

### Data collection and documentation

Trauma mechanisms were extracted from clinical records and categorized into predefined groups (assaults, falls, sports-related injuries, traffic accidents, iatrogenic causes, and others). Iatrogenic fractures were defined as fractures occurring as a direct consequence of medical or dental interventions. Bicycle-related injuries were classified as traffic accidents unless explicitly documented as sports-related. Preoperative DICOM datasets were reviewed using both the hospital’s PACS viewer (Sectra AB, Linköping, Sweden) and Horos v4.0.0 RC5™ (Horos Project, Annapolis, MD, USA). All three imaging planes (axial, coronal, sagittal) from CT and cone-beam CT (CBCT) scans were analyzed. Using the built-in measurement tools, we recorded the maximal cortical displacement of each fracture and the greatest shift of the mandibular canal, to one decimal place. To assess interrater reliability, 15 randomly selected DICOM datasets were independently remeasured by two experienced observers who were blinded to the clinical outcomes.

## Data preparation

All collected data were entered into a preformatted Microsoft Excel spreadsheet and pseudonymized in accordance with the General Data Protection Regulation. No patient-identifiable information was used during data processing or statistical analysis.

Continuous displacement measurements in axial, coronal, and sagittal planes (cortical and mandibular canal shifts in millimeters) were retained to one decimal place; binary indicators (“yes/no”) flagged the presence of any visible displacement. For overall CT analysis, the maximal displacement values in each anatomical direction were both kept as continuous variables and recoded into six ordinal categories (0.1–1.09, 1.1–3.09, 3.1–6.09, 6.1–9.0, > 9.0 mm) for standardized group comparisons. For overall CT analysis, total cortical displacement (“summed displacement”) was calculated as the simple sum of the maximal displacement values measured in the sagittal, transverse, and vertical planes. All other clinical parameters (demographics, comorbidities, injury etiology, peri- and postoperative data, and nerve function status) were similarly coded and validated prior to statistical analysis.

All data were cross-checked for plausibility and completeness prior to statistical evaluation.

### Statistical analysis

Statistical analysis was performed using IBM SPSS Statistics for Windows, version 28.0 (IBM Corp., Armonk, NY, USA). Categorical variables are presented as absolute and relative frequencies; continuous variables as mean ± SD. Group comparisons (e.g., presence vs. absence of preoperative IAN deficit) were undertaken using χ² tests for categorical data and ANOVA for continuous data.

To assess the reliability of the measurements obtained from the analysis of cortical displacement of the fracture in the individual imaging planes of the CT scans, the intraclass correlation coefficient (ICC) was calculated for each measured metric. For this purpose, 15 CT scans were randomly selected from the dataset of 237 evaluated CT images and remeasured independently at a later time point. The ICC model used was a two-way mixed-effects model with the definition of “absolute agreement.” [26].

Receiver operating characteristic (ROC) curve analysis determined optimal displacement cut-off values and areas under the curve (AUC). Univariable logistic regression models then evaluated independent predictors of IAN hypoesthesia (ORs with 95% CIs). All tests were two-sided, with *p* < 0.05 considered significant. Analyses were exploratory, and no correction for multiple comparisons was applied.

## Results

### Patient data

A total of 925 patients with mandibular fractures were identified (119 outpatient, 806 inpatient). After applying exclusion criteria, 258 inpatients met inclusion requirements, yielding 299 fracture events (including 41 patients with a second eligible fracture). Fifty patients were then excluded due to absent preoperative 3D imaging (34 underwent only 2D radiography; 16 lacked DICOM data), leaving 208 patients with 237 fractures for analysis – 29 of whom had contralateral injuries.

Among the 208 included patients, 43 (20.7%) were female and 165 (79.3%) male (M: F ratio 3.8:1). The mean age was 33.5 ± 16.8 years (median 28 years; range 9–89). Age distribution was 0–17 years (*n* = 16), 18–21 years (*n* = 41), 22–30 years (*n* = 60), 31–59 years (*n* = 73), and ≥ 60 years (*n* = 18). The mean BMI was 23.4 ± 3.9 kg/m² (range 14.6–38.8); five patients were underweight (< 18.5 kg/m²), 91 normal weight (18.5–25 kg/m²), and 31 overweight (> 25 kg/m²); BMI was unavailable for 81 cases. Epilepsy was documented in four patients (1.9%). Positive histories of smoking, alcohol abuse, and illicit drug use were recorded in 56, 36, and 10 patients, respectively.

### Preoperative clinical presentation and fracture classification

Among the 237 fractures, occlusal disturbances were noted in 55.7% (*n* = 132) of cases, while 41.4% (*n* = 98) had no malocclusion; 2.5% (*n* = 6) were edentulous, and one case lacked assessment. Restricted mouth opening occurred in 27.4% (*n* = 65) versus 72.6% (*n* = 172) without impairment. IAN hypoesthesia was documented in 31.2% (*n* = 74), absent in 65.0% (*n* = 154), and could not be assessed in 3.8% (*n* = 9) of cases.

Fracture sites among the 237 evaluable cases were classified as follows: mandibular angle (54.8%, *n* = 130), corpus (33.8%, *n* = 80), paramedian including mental-foraminal involvement (6.3%, *n* = 15), and comminuted fractures (5.1%, *n* = 12).

## Treatment course, complications, and recovery

The median interval from injury to surgery was 1 day (range 0–73 days; unknown in two cases); in 69 of 237 fractures (29.1%), treatment was delayed beyond 24 h, most often due to patient presentation delays, operating-room unavailability, or initial conservative management elsewhere. The mean postoperative inpatient stay was 3.2 days (range 0–33 days). Of the 237 fracture repairs, 235 were performed under general anesthesia and two under local anesthesia. Fourteen cases (5.9%) were managed with intermaxillary fixation alone.

Antibiotic prophylaxis was used adjunctively. An intravenous single-shot antibiotic dose was administered intraoperatively in 75.1% of patients (*n* = 178), predominantly with Unacid (89.9%), followed by cefuroxime (6.2%), penicillin V (2.3%), or clindamycin (1.7%). Postoperatively, 83.1% (*n* = 197) of patients received antibiotic therapy, and half of these continued antibiotics after discharge; amoxicillin/clavulanate was the most common agent post-discharge (65.3%), while Unacid remained the most commonly used during inpatient care (44.7%).

Postoperative complications occurred in 75 of 237 fracture repairs (31.6%). The most frequent finding was swelling with hematoma formation in 54 cases (22.8%). Among these, one patient developed a wound infection with dehiscence and an exposed osteosynthesis plate. No cases of restricted mouth opening were documented. Seven patients developed new postoperative occlusal disturbances, two of which remained unresolved despite intervention. Of the 75 complications, 11 were managed surgically, 48 conservatively, and 16 required no further treatment. In three of these cases, the sensory deficit had newly developed in the immediate postoperative period. The majority of IAN deficits resolved during follow-up, with only 3/237 fractures (1.3%) showing persistent hypoesthesia at ≥ 6 months.

## Injury etiology and mechanism

Overall, assaults were the leading cause of mandibular fractures (40.9%), particularly in young adult males (77.5% in ages 18–21 and 64.2% in ages 22–30); notably, 46.7% of male patients sustained fractures from assaults compared to only 7.0% of female patients. Falls (24.5%) were more frequent in elderly patients (66.7% of women ≥ 60 years and 50.0% of men ≥ 60 years). Sports-related injuries accounted for 10.1% of cases, affecting 19 men and two women. Iatrogenic fractures made up 6.3% of the cohort (*n* = 13) and occurred exclusively in adult male patients. The remaining 13.9% (*n* = 29) of fractures resulted from traffic accidents, work-related injuries, horse kicks, and suicide attempts. Detailed age- and sex-specific distributions are provided in Table 1.

**Table 1:** Gender-specific etiologies of mandibular fractures in the included and analyzed cohort

Female			Age groups									
			Children & adolescents (*n* = 3)		Young adults (*n* = 1)	Adults (*n* = 7)		Adults (*n* = 26)		Seniors (*n* = 6)		
			0–17		18–21	22–30		31–59		≥ 60		
Etiology		Assaults	0		1 (100.0%)	1 (14.3%)		1 (3.8%)		0		
		Falls	1 (33.3%)		0	4 (57.1%)		14 (53.8%)		4 (66.6%)		
		Sports-related injuries	0		0	0		2 (7.7%)		0		
		Traffic accidents	2 (66.7%)		0	0		4 (15.4%)		0		
		Iatrogenic injury	0		0	0		0		1 (16.7%)		
		Occupational accidents	0		0	0		0		1 (16.7%)		
		Horse kick injuries	0		0	2 (28.6%)		1 (3.8%)		0		
		Suicide attempts	0		0	0		1 (3.8%)		0		
		Unknown	0		0	0		3 (11.5%)		0		

**Male**			**Age groups**								
			Children & adolescents (*n* = 13)	Young adults (*n* = 40)			Adults (*n* = 53)		Adults (*n* = 47)		Seniors (*n* = 12)
			0–17	18–21			22–30		31–59		≥ 60
Etiology	Assaults		7 (53.8%)	31 (77.5%)			34 (64.2%)		9 (19.1%)		1 (8.3%)
	Falls		1 (7.7%)	3 (7.5%)			10 (18.9%)		8 (17.0%)		6 (50.0%)
	Sports-related injuries		3 (23.1%)	3 (7.5%)			5 (9.5%)		8 (17.0%)		0
	Traffic accidents		0	0			2 (3.7%)		5 (10.6%)		1 (8.3%)
	Iatrogenic injury		0	0			0		10 (21.3%)		2 (16.7%)
	Occupational accidents		0	1 (2.5%)			2 (3.7%)		5 (10.6%)		0
	Horse kick injuries		0	0			0		0		1 (8.3%)
	Suicide attempts		0	1 (2.5%)			0		0		0
	Unknown		2 (15.4%)	1 (2.5%)			0		2 (4.3%)		1 (8.3%)

Logistic regression analysis investigating additional influencing factors revealed that neither age nor sex had a statistically significant effect on the occurrence of hypoesthesia. Regarding the correlation between etiology and the likelihood of hypoesthesia, our analysis showed that occupational accidents were associated with a significantly increased risk – 6.3 times higher – compared to the reference group of sports-related injuries. Other causes of injury, such as falls, acts of violence, or iatrogenic factors, did not show a significantly higher risk compared to the reference group (Table 2).

**Table 2:** Logistic regression analysis investigating additional influencing factors

Variable	OR	95% CI	*p*-value^*^
Etiology (overall)			0.278
Acts of violence	2.533	(0.882; 7.277)	0.084
Falls	1.930	(0.622; 5.985)	0.255
Iatrogenic factors	1.867	(0.405; 8.614)	0.424
Occupational accidents	6.300	(1.275; 31.124)	0.024
Others	1.527	(0.340; 6.869)	0.581
Age	1.000	(0.984; 1.017)	0.966
Gender ♂	1.443	(0.681; 3.062)	0.339

### Predictive performance of displacement thresholds

Visible cortical displacement was common across all three CT planes. In the axial plane, 77.2% of fractures (*n* = 183) showed transverse cortical shift (mean 2.4 ± 0.2 mm), 92.4% (*n* = 219) a sagittal cortical shift (2.3 ± 0.2 mm), and 68.4% (*n* = 162) a mandibular canal shift (1.6 ± 0.2 mm). In the coronal plane, transverse cortical displacement occurred in 83.1% (*n* = 197; 2.4 ± 0.2 mm), vertical cortical displacement in 92.8% (*n* = 220; 2.2 ± 0.1 mm), and canal shift in 82.7% (*n* = 196; 2.0 ± 0.2 mm) of cases. Sagittally, cortical displacement was visible in 83.5% (*n* = 198; 2.5 ± 0.2 mm) and vertical cortical displacement in 76.8% (*n* = 182; 2.1 ± 0.2 mm), with a canal shift in 82.3% (*n* = 195; 1.9 ± 0.2 mm) (Table 3).

**Table 3:** Extent of cortical and inferior alveolar canal displacement on preoperative CT imaging in axial, coronal, and sagittal planes

Cortical displacement		Axial (*n* = 237)	Coronal (*n* = 237)	Sagittal (*n* = 237)
Parameter	Cortical transverse	77.2% (2.4 ± 0.2 mm)	83.1% (2.4 ± 0.2 mm)	83.5% (2.5 ± 0.2 mm)
	Cortical sagittal / vertical	92.4% (2.3 ± 0.2 mm)	92.8% (2.2 ± 0.1 mm)	76.8% (2.1 ± 0.2 mm)
	Mandibular canal displacement	68.4% (1.6 ± 0.2 mm)	82.7% (2.0 ± 0.2 mm)	82.3% (1.9 ± 0.2 mm)

Looking into these categories, the bulk of fractures demonstrated moderate displacement: vertical shifts fell into the categories 0.1–1.09 mm (31.2%) and 1.1–3.09 mm (32.9%), transverse shifts into 0.1–1.09 mm (26.2%) and 1.1–3.09 mm (30.0%), and sagittal shifts into 0.1–1.09 mm (27.4%) and 1.1–3.09 mm (39.2%). Minimal (0 mm) or extreme (> 9.0 mm) cortical displacement was uncommon (3.0–13.1% and 3.4–6.3%, respectively). Mandibular canal displacement similarly clustered in the categories 0.1–1.09 mm (38.0%) and 1.1–3.09 mm (27.8%), with fewer than 6% of cases in > 9.0 mm. These distributions provided the foundation for subsequent ROC analyses of each axis’ predictive power for nerve injury.

The reliability analysis of the measured cortical displacements in the different imaging planes yielded varying levels of agreement. The transverse displacement showed excellent reliability, with an ICC of 0.992 (95% CI: 0.978–0.997). The vertical displacement also demonstrated good reliability, with an ICC of 0.785 (95% CI: 0.382–0.927). In contrast, the sagittal displacement showed only average reliability, with an ICC of 0.573 (95% CI: -0.238–0.856). The displacement at the level of the mandibular canal was found to have excellent reliability as well, with an ICC of 0.944 (95% CI: 0.831–0.981).

### Receiver operating characteristic curve analysis and Youden index

**Fig. 1:**
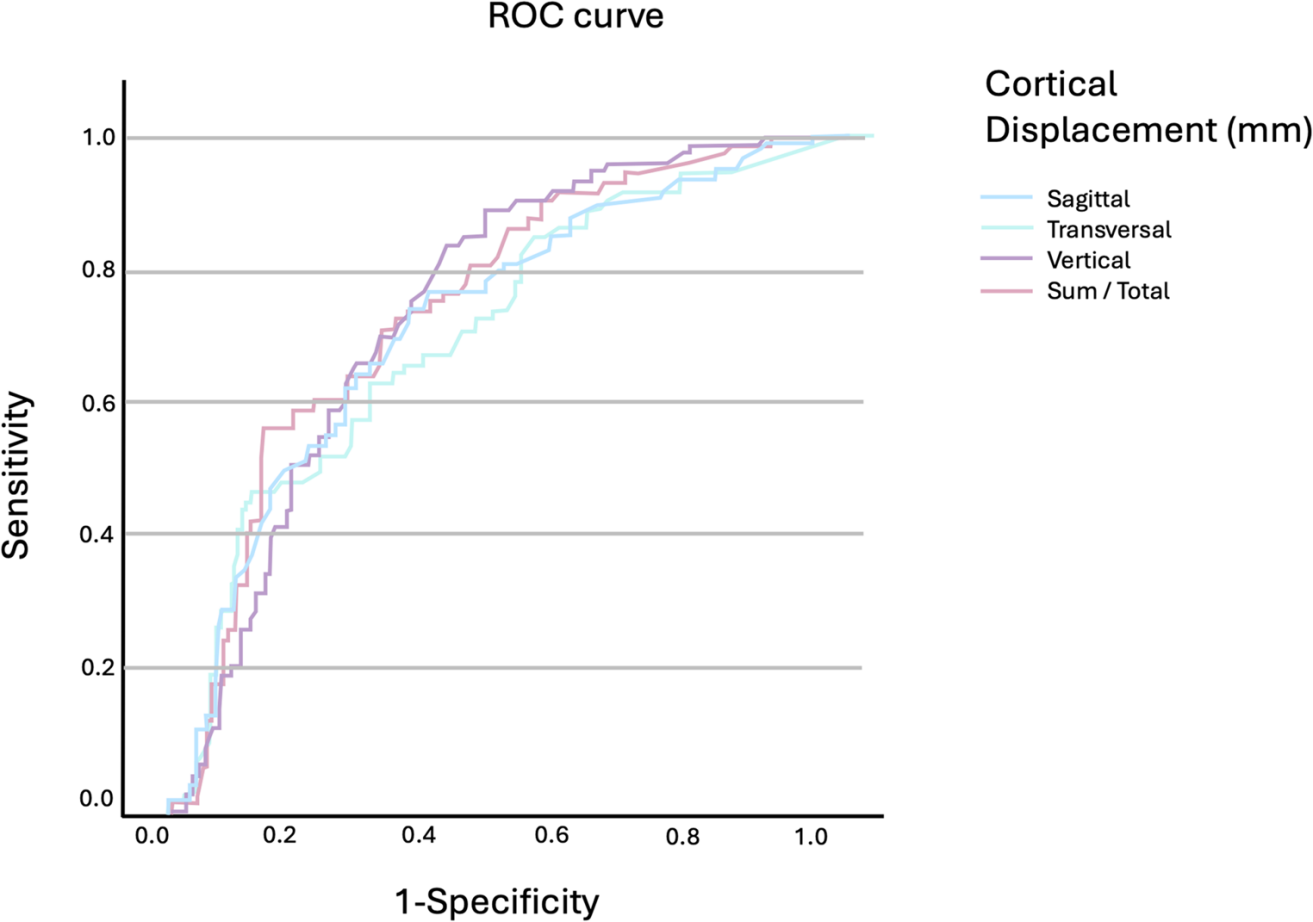
Receiver operating characteristic (ROC) curve analysis of cortical displacement in mandibular fractures (*n* = 237 fractures)

ROC analysis demonstrated good discrimination of inferior alveolar nerve hypoesthesia by cortical displacement thresholds (Fig. 1):

- Transverse shift > 3.60 mm: AUC 0.71 (95% CI 0.64–0.78; Youden 0.35).
- Sagittal shift > 1.68 mm: AUC 0.73 (95% CI 0.66–0.80; Youden 0.39).
- Vertical shift > 1.29 mm: AUC 0.75 (95% CI 0.69–0.81; Youden 0.43).
- Summed shift > 9.61 mm: AUC 0.75 (95% CI 0.69–0.82; Youden 0.43).

In univariable logistic regression, exceeding these cut-offs conferred significantly increased odds of preoperative hypoesthesia: transverse OR 6.4 (95% CI 3.3–12.4), sagittal OR 4.8 (2.6–8.9), vertical OR 6.8 (3.4–13.4), and summed OR 7.9 (4.1–15.0) (all *p* < 0.001). Multivariable models adjusted for age, sex, time to treatment, and etiology yielded similar effect sizes.

**Fig. 2:**
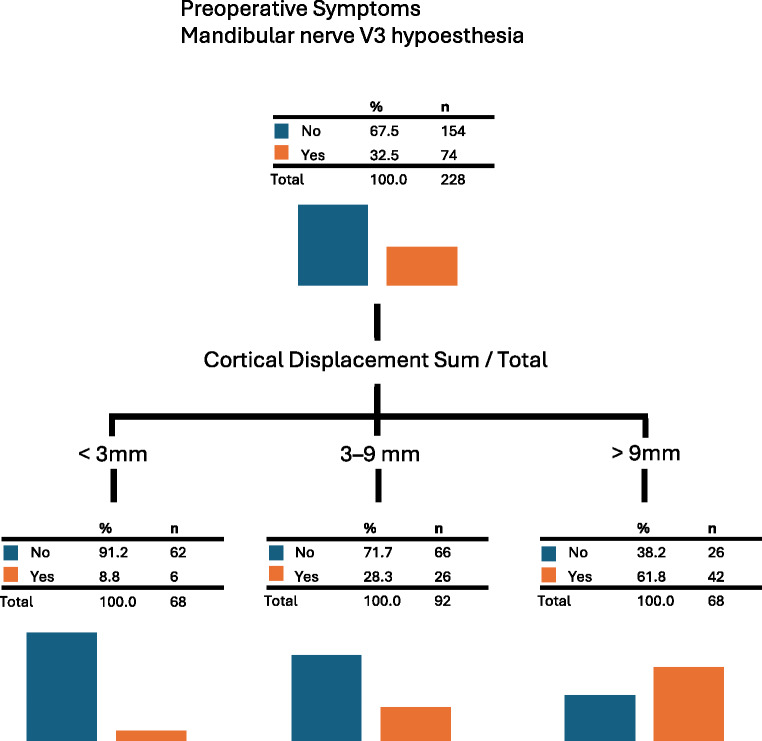
Decision-tree model for predicting preoperative inferior alveolar nerve (IAN) injury based on total cortical displacement (*n* = 228 fractures with assessable preoperative IAN status). The model stratifies patients into three risk groups: < 3 mm, 3–9 mm, and > 9 mm displacement

Decision-tree analysis (Fig. 2) based on total cortical displacement stratified preoperative IAN hypoesthesia into three distinct risk categories: < 3 mm, 3–9 mm, and > 9 mm. The corresponding incidences of hypoesthesia were 8.8% (6/68), 28.3% (26/92), and 61.8% (42/68), respectively, with an overall prevalence of 32.5% (74/228). The risk progression was monotonic – remaining low below 3 mm, increasing within the 3–9 mm range, and surpassing 60% beyond 9 mm. This stratification offers an intuitive and clinically applicable risk model that aligns with ROC-based threshold determinations.

**Fig. 3:**
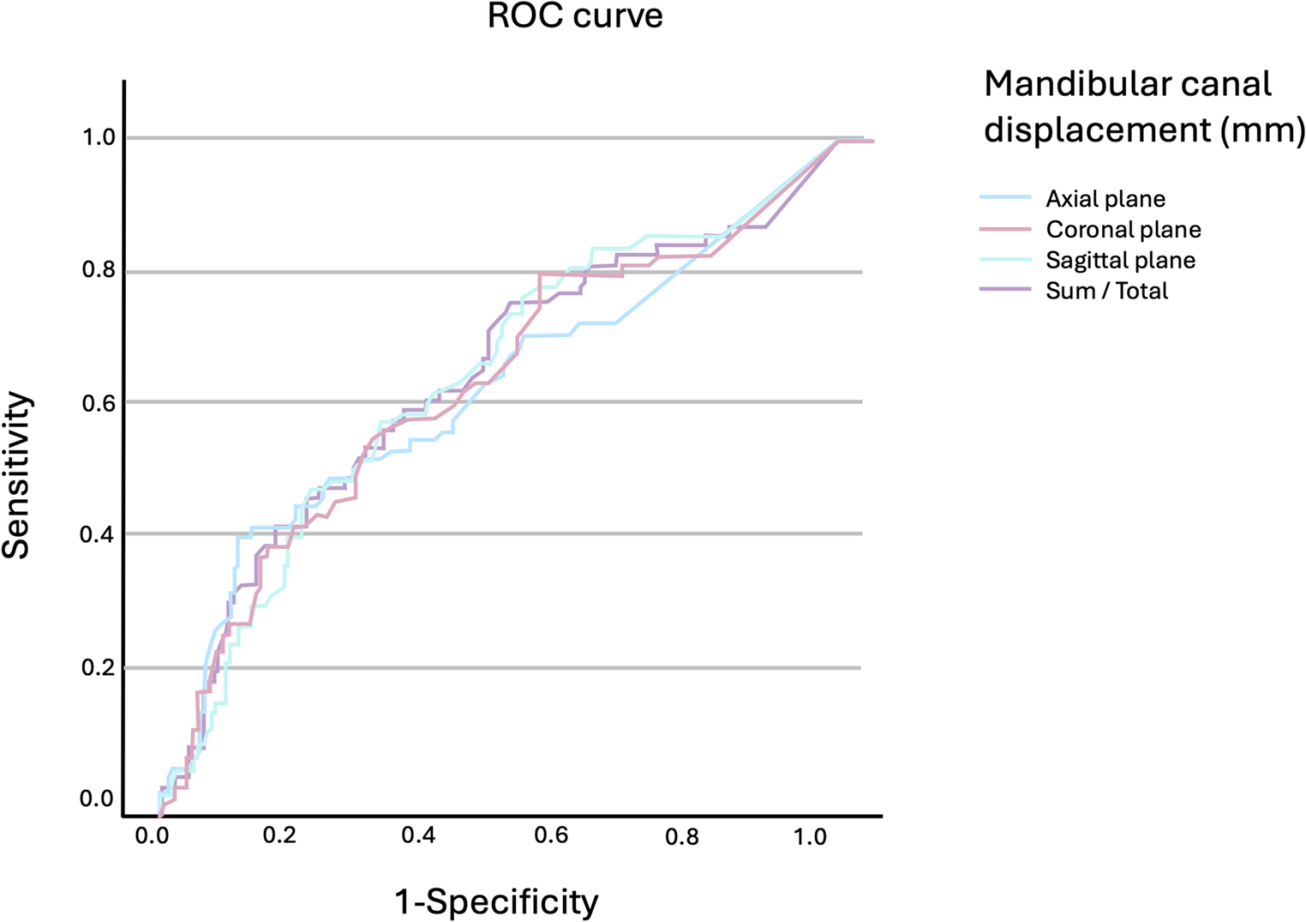
Receiver operating characteristic (ROC) curve analysis of mandibular canal displacement in mandibular fractures (*n* = 237 fractures)

Mandibular canal displacement showed limited predictive value for IAN hypoesthesia, with ROC AUCs of 0.625 (axial), 0.633 (coronal), 0.644 (sagittal), and 0.644 (summed displacement) (Fig. 3). Direct comparison demonstrated that cortical displacement thresholds consistently outperformed mandibular canal displacement as predictors of IAN hypoesthesia, with higher AUC values across all planes.

## Discussion

### Comparison with epidemiology and prior thresholds

Our cohort’s injury mechanisms align with established patterns: Assaults accounted for 40.9% of cases – well within the 28–84.5% range reported in previous series [10–12, 27] – while sports-related (10.1% vs. ~ 43%) and traffic-related fractures (~ 10% vs. 10–12%) were underrepresented compared to visceral cranial data [2, 3, 8, 28]. These differences reinforce the point that mandibular fractures have a distinct etiologic profile and justify the need for site-specific predictive tools.

Likewise, the displacement distribution in our overall CT analysis showed that minimal shifts (0 mm) were infrequent (3.0–13.1%) and extreme displacements (> 9 mm) occurred in only 3.4–6.3% of cases – suggesting that moderate cortical displacement is the norm and that risk stratification must focus on these intermediate ranges rather than extremes alone. The recorded preoperative symptoms – occlusal disturbances, restricted mouth opening, and inferior alveolar nerve hypoesthesia – closely mirror the classic clinical presentation of mandibular fractures reported in the literature [2, 3, 8, 28]. 

### Utility of the CT-based classification

A key novel aspect of this study is that, in contrast to previously reported thresholds derived primarily from two-dimensional panoramic radiographs, we provide the first systematic three-dimensional CT-based classification of fracture displacement for predicting IAN hypoesthesia. Our ROC-derived displacement thresholds (transverse > 3.60 mm, sagittal > 1.68 mm, vertical > 1.29 mm, summed > 9.61 mm) demonstrated strong predictive power (AUCs 0.71–0.75; OR 4.8–7.9), confirming that even small incremental shifts markedly increase IAN hypoesthesia risk (Fig. 1). Total cortical displacement (“summed displacement”) emerged as a key variable for stratifying the risk of preoperative IAN hypoesthesia. Through decision-tree analysis (Fig. 2), we derived a simple and clinically applicable three-tier classification: < 3 mm, 3–9 mm, and > 9 mm. This stratification was associated with increasing rates of hypoesthesia – 8.8% (6/68), 28.3% (26/92), and 61.8% (42/68), respectively – with an overall incidence of 32.5% (74/228). The risk gradient observed was monotonic, with a clear escalation in probability across the defined thresholds. We emphasize the practicality of this straightforward categorization, which facilitates intuitive preoperative risk assessment and aligns well with ROC-based threshold determinations.

In contrast, mandibular canal displacement performed poorly as a standalone predictor of IAN hypoesthesia. ROC analysis yielded AUCs of 0.625 (axial), 0.633 (coronal), 0.644 (sagittal), and 0.644 (summed displacement), all below the accepted threshold for useful discrimination (Fig. 3). In other words, canal shift alone cannot reliably distinguish between patients with and without preoperative nerve deficits. A subsequent classification-tree analysis did identify a canal displacement > 4.1 mm as the point at which the probability of hypoesthesia reached ≥ 50%, but overall, these findings suggest that cortical displacement metrics are markedly superior for predicting IAN hypoesthesia. This association is biomechanically coherent, as the extent of nerve elongation is dependent on the axis of rotation, which in turn affects nerve strain proportionally to the square of its distance from the nerve. Nonetheless, these findings contrast with those of a study by Sahoo et al., who analyzed 72 fractures. Their results suggest a higher incidence of paresthesia in patients with mandibular canal discontinuity, although the interpretation is limited by the relatively small sample size [29]. By categorizing fractures into standardized CT-based displacement groups, clinicians can more accurately counsel patients on nerve-injury probability, tailor surgical approaches, and, in forensic contexts, provide objective thresholds for evaluating permanent sensory deficits.

Beyond the plane-specific thresholds, the comprehensive assessment of total cortical displacement (“summed displacement”) further integrates the diagnostic potential of modern multiplanar CT imaging, which has become standard in clinical practice. Such an approach to evaluating displacement represents a logical extension of modern CT-based trauma assessment and, in our findings, demonstrates the highest predictive value for the presence of initial inferior alveolar nerve (IAN) injury. The highest risk for preoperative hypoesthesia was associated with the statistical parameter of total cortical displacement, showing a 7.9-fold increase (*p* < 0.001). A vertical cortical displacement greater than 1.29 mm also demonstrated a highly significant 6.8-fold increased risk for the presence of preoperative hypoesthesia (*p* < 0.001). Similarly, transverse cortical displacement over 3.6 mm was associated with a 6.4-fold significantly increased risk of preoperative hypoesthesia (*p* < 0.001). From a clinical perspective, fractures exceeding the > 9 mm displacement threshold represent a high-risk group for IAN hypoesthesia. In these cases, clinicians should anticipate a substantially increased likelihood of preoperative hypoesthesia and consider this in surgical planning, for example by prioritizing early reduction, careful handling of the neurovascular bundle, and thorough preoperative patient counseling regarding the elevated risk of persistent sensory deficits. In addition, the proposed CT-based classification provides an objective and reproducible framework that may support medico-legal assessments, particularly in cases where the likelihood of permanent nerve injury and its causal relationship to the initial trauma versus surgical intervention must be evaluated.

### Recovery

A large majority of patients experienced complete resolution of IAN hypoesthesia by six months post-injury, with only three individuals (1.3% of fractures) reporting persistent sensory deficits, underscoring the nervous system’s inherent capacity for gradual functional recovery over time. This excellent recovery trajectory aligns with previous reports, where most IAN disturbances following mandibular fracture gradually resolved within 6–12 months [13, 17, 19]. 

Considering the applied exclusion criteria, a good homogenization of our study cohort was achieved. A rate of persistent hypoesthesia of 1.3% is consistent with findings in the existing literature and reflects an overall high standard of clinical care. In well-reduced fractures, the mandibular canal provides an optimal boundary for nerve regeneration [24, 30, 31]. Due to the retrospective study design, the extent of hypoesthetic areas and further functional limitations experienced by the affected patients could not be evaluated. However, this aspect is intended to be addressed in a planned prospective study, which will apply our CT-based categorization to more precisely assess the extent and impact of sensory impairment.

Another factor contributing to the generally favorable outcomes with a low rate of major complications in this study was the prompt treatment of patients within 24 h. Hassan et al. demonstrated a correlation between the timing of the fracture occurrence and the initiation of treatment [32]. This association was also described by Tabrizi et al., who reported that early open reduction with internal rigid fixation following mandibular fractures appears to shorten the recovery time of neurosensory disturbances of the IAN in cases of mandibular body fractures [33]. 

### Strengths, limitations, and future research

This study’s strengths include a large, single-center cohort and rigorous, plane-specific CT measurements with good to excellent interrater reliability.

This study has several limitations. Due to the retrospective design, the study is prone to inherent biases and missing data; in particular, a detailed, standardized evaluation of the extent and functional impact of hypoesthesia was not feasible. Furthermore, the duration and completeness of follow-up may have been insufficient to capture all cases of late or persistent hypoesthesia, potentially leading to an underestimation of long-term sensory deficits. In addition, surgical technique and operator-dependent factors (e.g., fixation method, surgical experience, and intraoperative handling of the neurovascular bundle) were not controlled for, which may influence postoperative nerve outcomes. Additionally, persistent hypoesthesia may not only be a consequence of the initial injury but can also result from iatrogenic nerve damage, particularly when the IAN is affected during open reduction and internal fixation (ORIF). Distinguishing between injury- and surgery-related hypoesthesia is challenging and represents a potential confounding factor. Addressing this issue reliably in future prospective studies will likely require large patient cohorts drawn from high-volume centers, where surgical procedures and outcomes can be more systematically analyzed. The findings require prospective validation to confirm generalizability and to refine displacement categories – especially around intermediate thresholds – for optimized risk stratification. Future research should also explore integrating CT displacement with other risk factors (e.g., time to treatment, patient comorbidities) to develop comprehensive predictive models for IAN hypoesthesia.

Despite these limitations, the present study provides valuable insights into predictive factors for nerve injury following mandibular fractures.

## Conclusion

Our CT-based classification, using summed cortical displacement thresholds (< 3 mm, 3–9 mm, and > 9 mm), reliably stratifies the risk of IAN hypoesthesia in mandibular fractures. By integrating these quantitative criteria into preoperative assessment, clinicians and medico-legal experts can enhance surgical planning, improve patient counseling on nerve-injury likelihood, and provide standardized justification for expert opinions on permanent sensory deficits. 

## Data Availability

No datasets were generated or analysed during the current study.

## Abbreviations

AUC: Area under the (ROC) curve
ANOVA: Analysis of variance
CT: Computed tomography
DICOM: Digital Imaging and Communications in Medicine
CBCT: Cone-beam computed tomography
IAN: Inferior alveolar nerve
ICD-10-GM: International Classification of Diseases, 10th Revision, German Modification
OR: Odds ratio
PACS: Picture archiving and communication system
ROC: Receiver operating characteristic
SD: Standard deviation
χ² test: Chi-square test

## Declarations

### Clinical trial

This manuscript does not report on a clinical trial.
